# Supplementary material for: Probing the composition dependence of residual stress distribution in tungsten-titanium nanocrystalline thin films
Source: Commun Mater. 2023 Feb 15;4(1):11. doi: 10.1038/s43246-023-00339-6 (PMC11041690; doi:10.1038/s43246-023-00339-6)
Supplement: Supplementary file 1 — Supplementary Information [file 43246_2023_339_MOESM1_ESM.pdf]

Supplementary Information:

Probing the composition dependence of residual stress distribution in tungsten-titanium nanocrystalline thin films

Rahulkumar Jagdishbhai Sinojiya[1], Priya Paulachan[1], Fereshteh Falah Chamasemani[1], Rishi Bodlos[1], René Hammer[1], Jakub Zálešák[2], Michael Reisinger[3], Daniel Scheiber[1], Jozef Keckes[2], Lorenz Romaner[2], Roland Brunner[1]

[1]Materials Center Leoben Forschung GmbH, A-8700-Leoben, Austria.

[2]Department of Materials Science, Montanuniversität Leoben, A-8700 Leoben, Austria,

[3]KAI Kompetenzzentrum Automobil - u. Industrieelektronik GmbH, A-9524 Villach, Austria

Email: roland.brunner@mcl.at

SUPPLEMENTARY FIGURES:

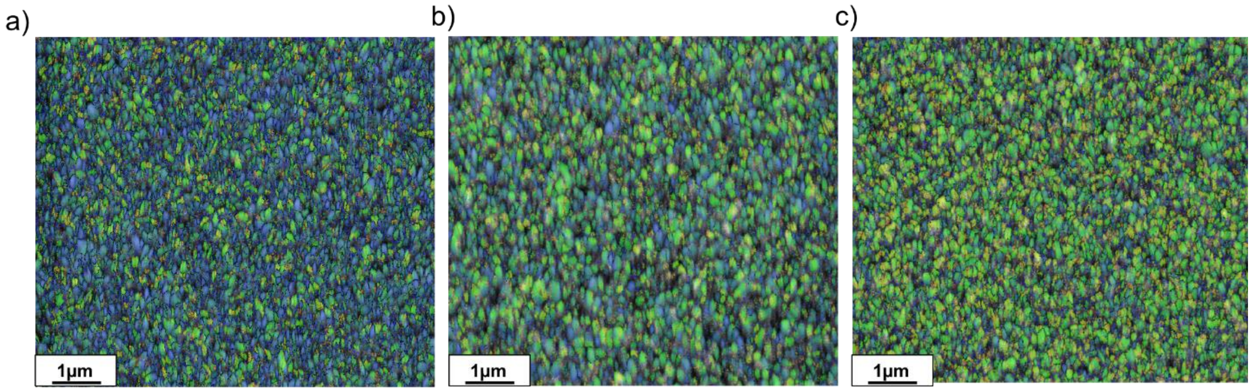

Supplementary Figure 1| Misorientation map for $W_{1-x}Ti_x$ thin films on surface: Misorientation map is plotted for the (a) 30 at% Ti, (b) 20 at% Ti and (c) 15 at% Ti. AztecCrystal software is used to plot the misorientation maps. It shows that the 30 at% Ti has the highest misorientation while 15 at% Ti has the lowest.

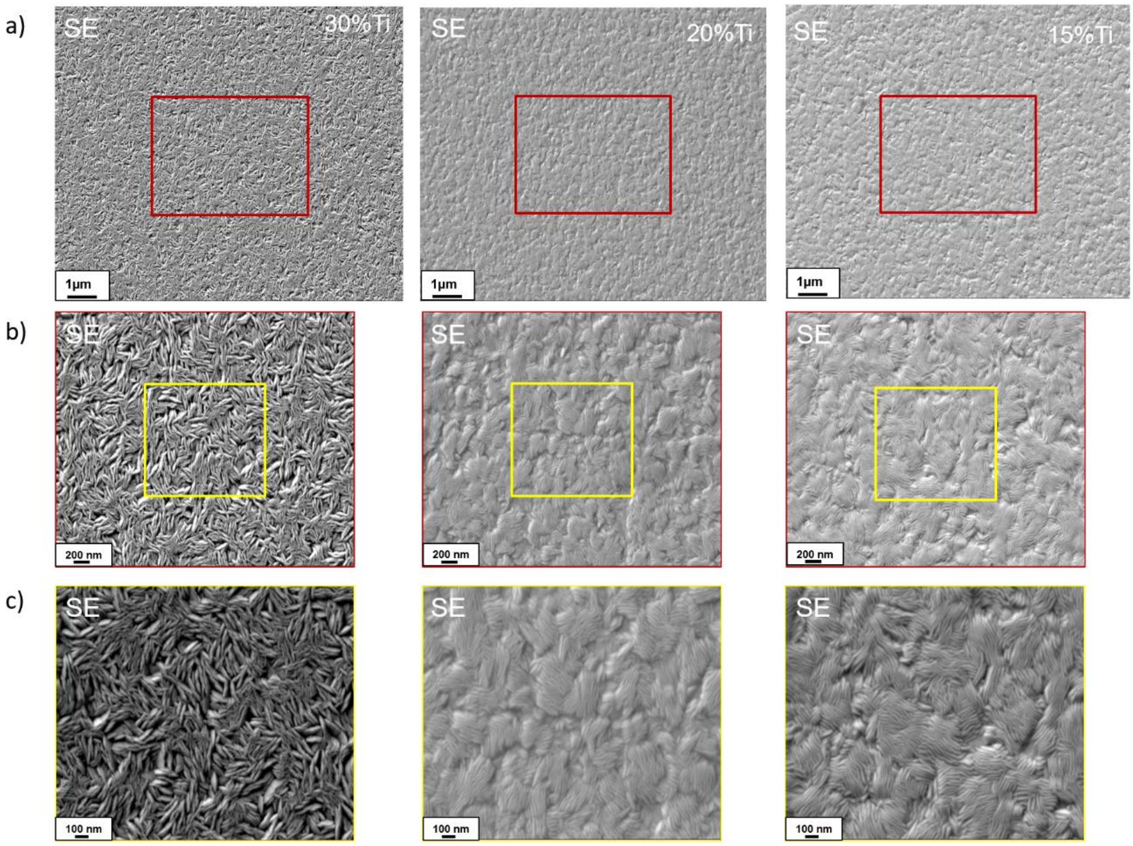

Supplementary Figure 2| SEM Grey scale images: In this figure, SE-SEM images are shown for the $W_{1-x}Ti_x$ thin films at different magnifications and scale. (a) SE-SEM images at 1µm scale are shown for 30 at% Ti, 20 at% Ti and 15 at% Ti respectively. (b) SE-SEM images at 200nm scale for 30 at% Ti, 20 at% Ti and 15 at% Ti respectively. (c) SE-SEM images at 100nm scale for 30 at% Ti, 20 at% Ti and 15 at% Ti respectively.

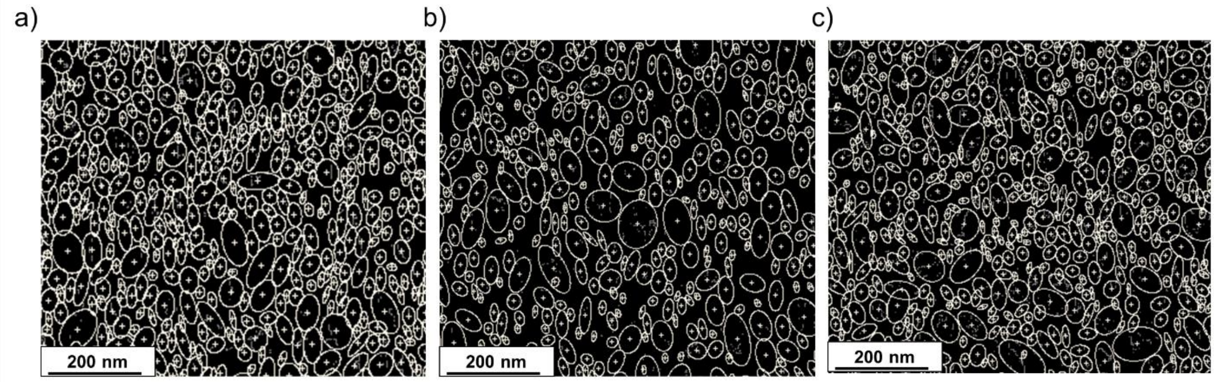

Supplementary Figure 3| Ellipsoids fitted on surface for grains: Ellipses are fitted to the grains respectively for (a) 30 at% Ti, (b) 20 at% Ti and (c) 15 at% Ti (left to right). These ellipses are plotted using the ellipse fitting algorithm in AztecCrystal and justifies the shape of the grains w.r.t the statistical analysis shown in Table 1 in main manuscript.

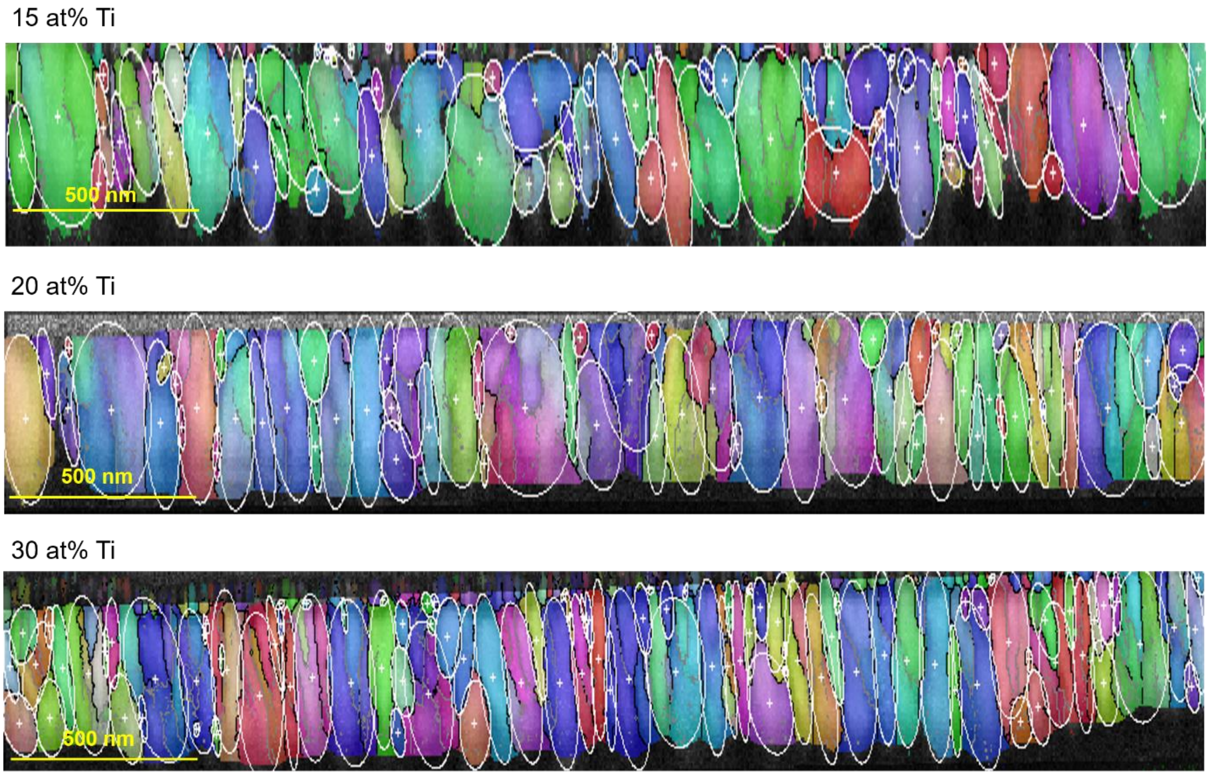

Supplementary Figure 4| Cross sectional EBSD (Reflective mode) showing the fitted ellipsoids to the grains for 15 at% Ti, 20 at% Ti and 30 at% Ti, respectively.

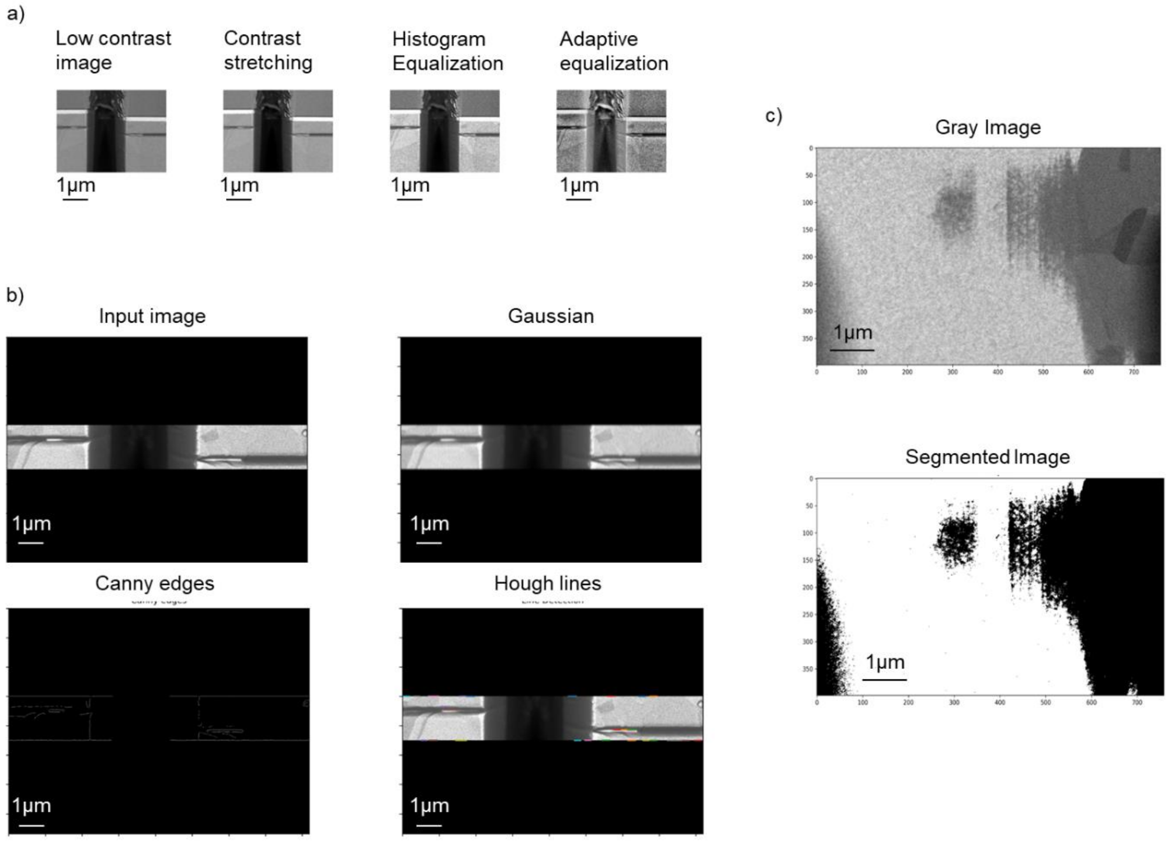

Supplementary Figure 5| Image analysis-based deflection profile extraction. Conventional image analysis. (a) Preprocessing of the raw image from SEM. First contrast stretching is performed and then based on grey values, we do histogram equalization for the images to make it to the similar grey values. (b) A Gaussian blur is applied to the equalized image followed by the canny edge detection. Hough transform is applied to detect lines from canny edge detected image. These detected lines help us to estimate the deflection for the given step from ILR method. (c) To identify the interface between several materials in a multi material stack configuration, binary segmentation is applied to evaluate the exact interface distance. This helps to understand the deflection profile classified in the different thin film layer.

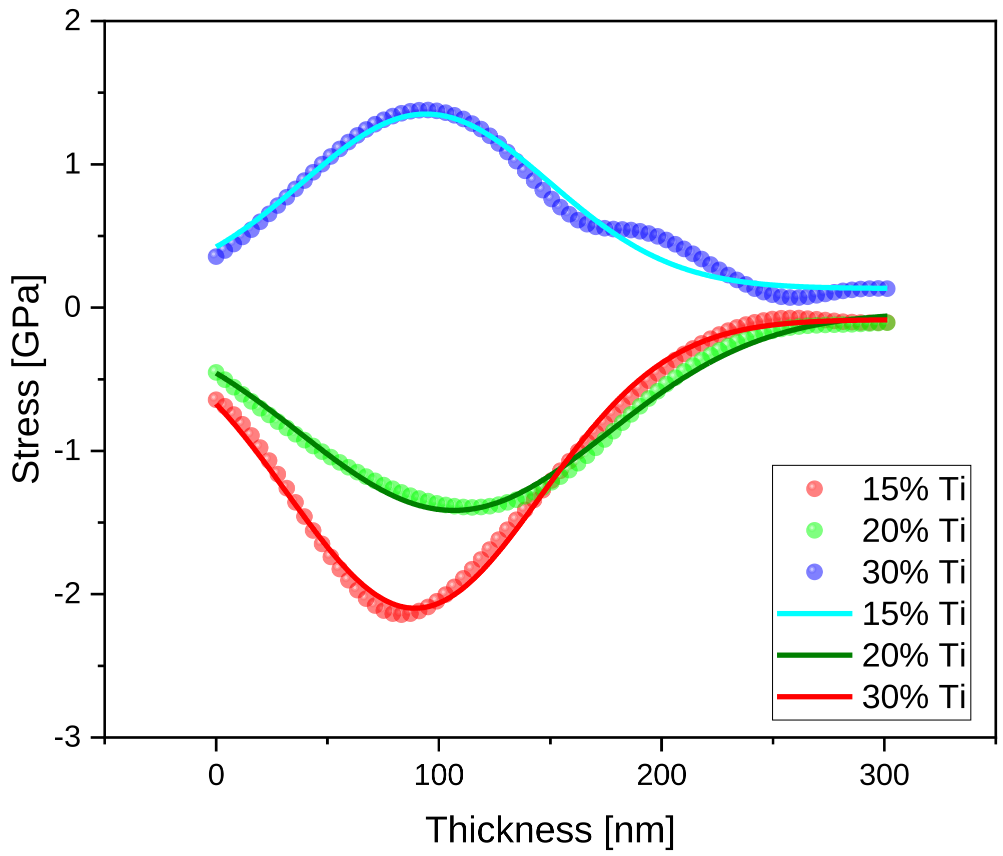

Supplementary Figure 6| Gaussian fit FWHM in stress profiles: A gaussian fit is applied to the residual stress profiles. The fitted lines are shown above the actual stress values. Red for 15 at% Ti, green for 20 at% Ti and blue for 30 at% Ti is plotted.

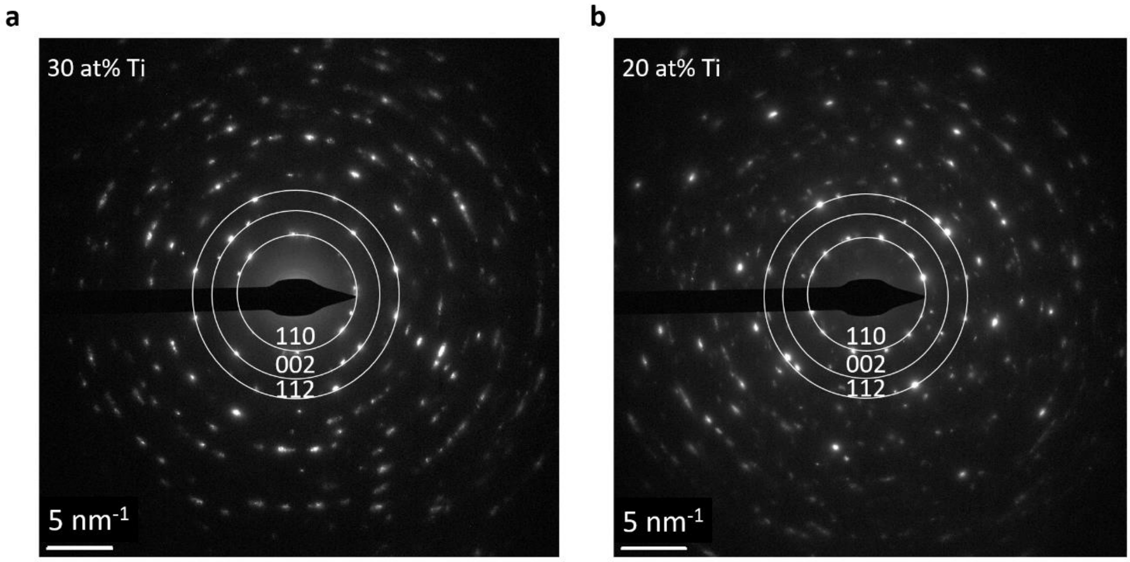

Supplementary Figure 7| STEM diffraction results a Orientation map for 30 at% Ti **b** Orientation map for 20 at% Ti.

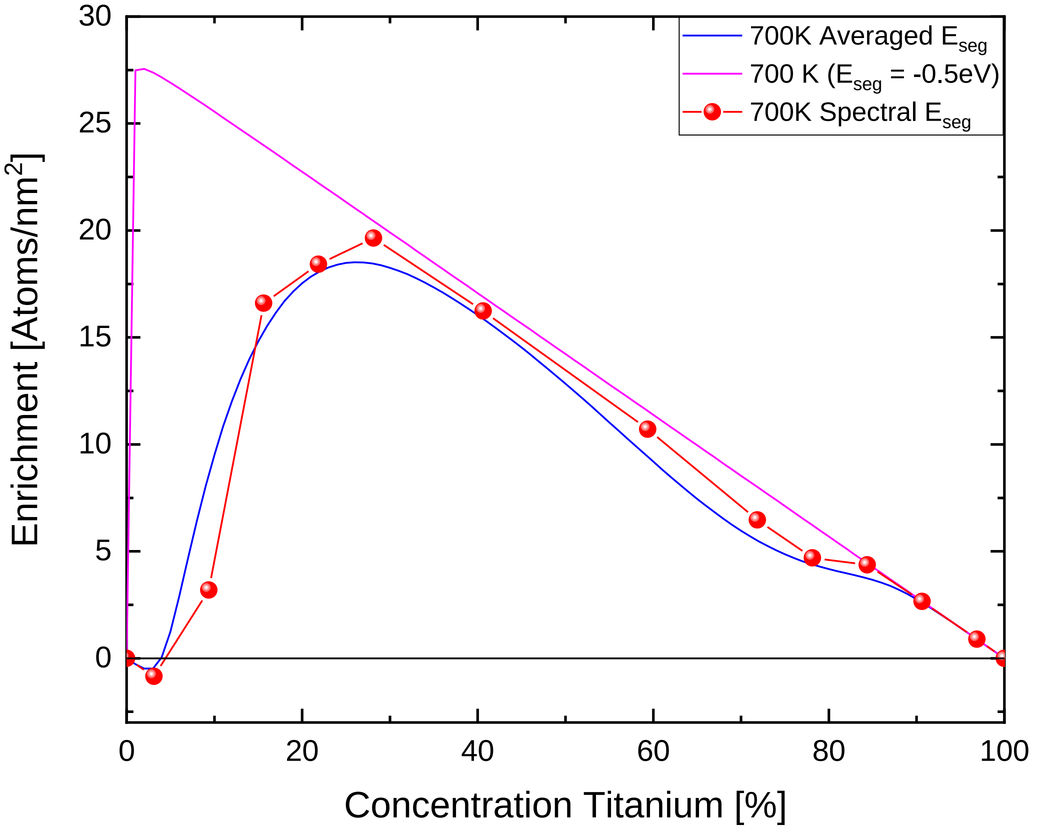

Supplementary Figure 8| Ti Enrichment of the GB when using Averaged E_{seg}, Spectral E_{seg} and a segregation energy of -0.5eV at 700 K temperature.

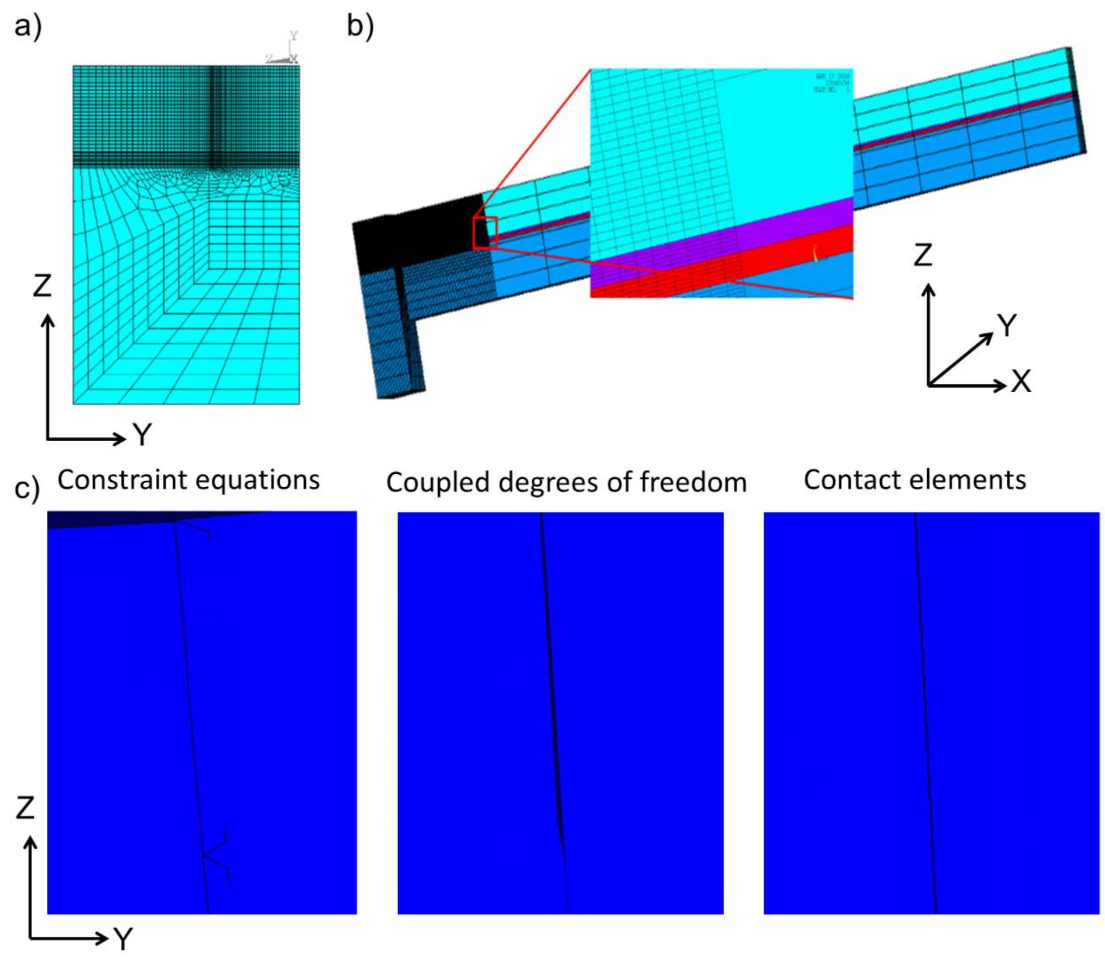

Supplementary Figure 9| Meshing FEM model using Contact Elements: (a) A 2d mesh is shown at the interface from which a 3d volumes will be extracted. ILR region is meshed finely and then gradually mesh becomes coarser towards the end support. (b) A finished 3D model is shown with the zoomed mesh difference at the interface. (c) Interface meshing is shown using constraint equations, coupled degree of freedom and contact elements method. The best result is obtained using contact elements (area to area) as the volumes are not overlapped or underlapped.

Supplementary Note 1: Image analysis (segmentation)

The images of size 3 x 2 μm^2 were used for analyses of each wafer. To determine the orientation of needles a multi-step image analyses was performed. First, the median filter was applied to alleviate the noise. Then, the local threshold was applied to the pre-processed images to segment the needles. In the next step, the watershed algorithm was used ahead of calculating the needles orientation. Finally, the lowest intensity was assigned to the background and the calculated orientation of each needle was replaced with its defined labelled in each image. We carried out the analyses using Python libraries including Numpy, Scipy, Scikit-image. The wafers orientation was visualized using Avizo® (version 2020.2).

Supplementary Note 2: Image analysis based deflection extraction

Once the ILR is performed, the image based raw data (Fig.2 in main text) is received for image analysis. Image analysis is performed for removed layer thickness and deflection measurement. The reason of doing image analysis in python is to automatize the method. Image analysis helps in reducing time and human errors for analysis of data.

As shown in Supp. Fig 5a, histogram equalization is performed as the first step to make all the images on the same gray scale. If not performed, different images might need different parameters for further analysis and the method can't be automatized. Gaussian blur is implemented to remove noise from the images which helps in better edge and line detection. As shown in Fig. 2 (main text), the deflection marker is at the tip of the cantilever, which is used to calculate deflection from image analysis. This reference marker is detected by line detection. To perform efficient line detection, Gaussian blur is implemented. Once blur is applied, canny edge detection is applied to detect edges around the deflection marker. Then detected edges are used in Hough transformation to detect lines around the deflection marker. Lines are detected around the deflection marker. The number of pixels in the give image is calculated as a result of the difference in the detected lines of the beam and the reference marker on the fixed side of the beam. Using the image pixel size and the number of pixels, deflection for the single sublayer step is calculated. This process is automatized for all the images for a sample. Therefore, to measure the layer thickness same procedure is carried out with the difference of line detection position. In the end of image analysis, we obtain the deflection profile as a function of layer thickness. This deflection profile is then used to calculate the stresses analytically as well as finite element simulations.

Supplementary Note 3: DFT

For DFT calculations we used the Vienna ab initio simulation package with periodic boundary conditions and the projector augmented wave method (PAW)[1–4]. The detailed methodology used for the DFT calculations of Bulk WTi is given in Ref[5]. For the grain boundary calculations we employed the same input parameters as for the bulk calculations with the cells shown in main text Figure 5a.

We calculated segregation energies in the dilute limit according to:

$$E_{seg} = \left(E_{GB}^{nTi} - E_{GB}^{(n-1)Ti}\right) - \left(E_{B}^{1Ti} - E_{B}^{0Ti}\right) \tag{7}$$

With GB and B standing for the total energy from a grain boundary and bulk cell, respectively, and the super script denotes the number of Ti atoms in the cell. For higher Ti bulk concentrations, the segregation energy was calculated from the formation energies as

$$E_{seg} = \frac{\partial E_{form}^{GB}}{\partial c_{Ti}^{GB}} - \frac{\partial E_{form}^{B}}{\partial c_{Ti}^{B}} . \tag{8}$$

The formation energy is usually defined as

$$E_{form}^{Bulk} = \left(E_{tot} - \sum_i E_{ref}^{i}\right)/N \tag{9}$$

where E_{tot} is the total energy of an atomic compound (bulk, GB, or other structure), E_{ref}^{i} is the bulk reference energy of element i in its ground state, and N is the number of atoms in the compound. So the formation energy is the energy it takes to assemble a specific atomic compound from its elemental ingredients. The GB is a somewhat special case as it is an extended defect where periodic boundary conditions enforced in the DFT calculations have to be considered. For the chosen GB setup with a GB slab that is separated on the opposite ends (vacuum on top setup), the formation energy for the GB reads

$$E_{form}^{GB} = \left[E_{tot}^{GB,N_{Ti}} - E_{tot}^{FS,W} - N_{Ti}\left(E_{ref}^{Ti} - E_{ref}^{W}\right)\right]/N. \tag{10}$$

Here, $E_{tot}^{GB,N_{Ti}}$ is the total energy of a cell containing a slab with a GB and two surfaces with N_{Ti} Ti atoms at the GB, whereas $E_{tot}^{FS,W}$ is the total energy of a similar slab but without a GB and containing only W atoms. For $N_{Ti} = 0$, the first two terms already constitute the formation energy of the GB. The last term gives the correction in case the GB contains Ti atoms by the reference energies for Ti and W, respectively. The formation energy is then normalized by the number of atoms in the GB, which in our case are all atoms in the central GB layer and the two layers next to it on both sides of the GB. The segregation energy obtained from the formation energies is an averaged quantity as the formation energies are already an averaged quantity.

Based on the segregation energies and the Ti concentration in the bulk, the concentration at the GB can be computed as a function of the temperature using the McLean isotherm[6]:

$$\frac{c_{Ti}^{GB}}{1-c_{Ti}^{GB}} = \frac{c_{Ti}^{B}}{1-c_{Ti}^{B}} \exp\left(\frac{-E_{seg}}{k_B T}\right) \tag{11}$$

The GB enrichment or interfacial enrichment (IFE) is then computed as

$$IFE = n_{GB}(c_{Ti}^{GB} - c_{Ti}^{B})/A \tag{12}$$

With A the area of the GB and n_{GB} the number of atoms in the GB. This quantity denotes how much more Ti atoms are present at the GB in comparison to the bulk. If the segregation energy depends on the GB concentration, i.e. $E_{seg}\left(c_{Ti}^{GB}\right)$, this equation needs to be solved iteratively. Note that for reasons of simplicity, this aspect has been usually neglected in most DFT investigations of GB segregation so far. Another simplification often encountered is the assumption that there is only one average segregation energy for the whole GB. In reality the GB structure is made up of multiple distinctive sites with different segregation energies (see Fig. 5a) and for an accurate treatment, the spectral nature of the segregation energy needs to be considered[7,8]. This can be achieved by adding an index i for the GB sites so that we have a specific segregation energy and GB concentration at each site, E_{seg}^{i} and $c_{Ti}^{GB,i}$. The overall concentration at the GB is computed by averaging the site concentrations at the GB. A comparison of the

approach with spectral segregation energies and averaged segregation energy is given in Supp. Fig. 8, which shows that in this specific case, the effect is negligible, as long as the dependency of the bulk concentration is taken into account.

Supplementary Note 4: Meshing FEM model using Contact Elements and Optimisation Routine

As explained before, the region of interest is the ILR region, so in the corresponding volume the model is meshed with very small element size as shown in supp Figure 9. The element size with respect to the layer normal has to be at least as small as the dimensions of the individual slices removed by the corresponding ILR step. Hence, our focus is to reduce number of elements to reduce the excessive computational time which would be the result if the cantilever would be meshed with this fine mesh everywhere. One crucial observation is that the rest of the beam is unaltered when the ILR is performed in the ILR region only (that means the curvature in the remaining part remains frozen). Therefore, the cantilever support as well as the region acting as an indicator like rigid body can be meshed with a very coarse mesh. A non-conformal mesh is used to allow a strong transition from the fine mesh in the ILR region to the coarse mesh regions. Different methods are available to connect these regions. One is to generate constraints equations at an interface, but this method leads to define more equations than usual causing the increase in computation time. Another way is to define coupled degrees of freedom at an interface, but this only couples the nodes and the volumes are getting either overlapped or underlapped at the interface. The third method is to use contact elements area to area. It turned out that this pragmatic third approach provided the best results in terms of computational efficiency and minimization of solution artefacts near the boundary. Once the meshing is completed, a rigid body is defined with all the boundary conditions to make the model as realistic as possible.

A Regula-Falsi method commonly known as bisectional method / false position method is used for optimization. It start with two points, (a,f(a)) and (b,f(b)), satisfying the condition that f(a)f(b) < 0. The straight line through the two points (a,f(a)), (b,f(b)) is

$$y = f(a) + \frac{f(b)-f(a)}{b-a}(x-a), \tag{4}$$

The next approximation to the zero is the value of x where the straight line through the initial points crosses the x-axis.

$$\mathrm{x} = a - \frac{b-a}{f(b)-f(a)}, \tag{5}$$

$$f(a) = \frac{af(b)-bf(a)}{f(b)-f(a)}, \tag{6}$$

In this method an interval of the stress value is given as an input and the new values of these intervals is calculated until tolerance is met. This method is implemented in Python. To implement this with ANSYS, a subroutine is called in python to run ANSYS and extract the results and compare back in Python. In order to reduce the computational effort in the model the procedure operates in with respect to the experiment in the opposite time direction. This means, that the first step in the simulation is adding the first slice (which was removed last in the experimental ILR procedure). This has the advantage that one starts from a known system (substrate only) and only one stress value has to be optimized to match the experimentally observed deflection at each step. The optimized stress value for this slice is then already known and used when the next slice is evaluated afterwards.

	TiW	Minor axis of ellipse	Minor axis of ellipse	Aspect ratio of fitted ellipse	Aspect ratio of fitted ellipse	Grain density	Area of the ellipses
	Ti%	nm	nm			µm^{-2}	x 10^3 nm^2
		Mean	Standard deviation	Mean	Standard deviation		
TKD Cross section	15	31	56	2.68	1.16	178	8.1
	20	34	46	2.92	1.98	120	11.1
	30	27	25	3.37	1.17	252	5.2
Cross section	15	52	48	1.95	0.84	68	3.8
	20	64	50	2.47	1.15	41	7.3
	30	40	62	2.66	1.23	109	3.4

Supplementary Table 1: FESEM-EBSD cross section (x-z plane) and FESEM-TKD cross section (x-z) grain analysis illustrating the mean value and standard deviation for minor axis, aspect ratio and area of the fitted ellipses as well as the grain density.

Supplementary References:

1. Kresse, G. & Hafner, J. Ab initio molecular-dynamics simulation of the liquid-metal--amorphous-semiconductor transition in germanium. *Phys. Rev. B* **49**, 14251–14269 (1994).
2. Kresse, G. & Furthmüller, J. Efficiency of ab-initio total energy calculations for metals and semiconductors using a plane-wave basis set. *Comput. Mater. Sci.* **6**, 15–50 (1996).
3. Blöchl, P. E. Projector augmented-wave method. *Phys. Rev. B* **50**, 17953–17979 (1994).
4. Joubert, D. From ultrasoft pseudopotentials to the projector augmented-wave method. *Phys. Rev. B - Condens. Matter Mater. Phys.* **59**, 1758–1775 (1999).
5. Bodlos, R. *et al.* Ab initio investigation of the atomic volume, thermal expansion, and formation energy of WTi solid solutions. *Phys. Rev. Mater.* **5**, 1–10 (2021).
6. P. Lejček. *Grain Boundary Segregation in Metals*. (Springer Verlag, Berlin, 2010).
7. Huber, L., Hadian, R., Grabowski, B. & Neugebauer, J. A machine learning approach to model solute grain boundary segregation. *npj Comput. Mater.* **4**, 64 (2018).
8. Scheiber, D., Jechtl, T., Svoboda, J., Fischer, F. D. & Romaner, L. On solute depletion zones along grain boundaries during segregation. *Acta Mater.* **182**, 100–107 (2020).
